# Experiments to Determine the Disinfecting Value of the Morris Formaldehyde Generator*Now called the Germicidal Gas Generator.—Editor Journal.

**Published:** 1903-08

**Authors:** Allen J. Smith

**Affiliations:** Professor of Pathology; Bacteriologist of the Medical Department, University of Texas, Galveston, Texas


					﻿THE
TEXAS MEDICAL JOURNAL.
ESTABLISHED JULY, 1885.
PUBLISHED MONTHLY.—SUBSCRIPTION $1.00 A YEAR.
Vol. XIX.
AUSTIN, AUGUST, 1903.
No. 2.
Original Contributions.
Experiments to Determine the Disinfecting Value
of the Morris Formaldehyde Generator.*
[*Now called the Germicidal Gas Generator.—Editor Journal.]
BY ALLEN J. SMITH, M. D.,
Professor of Pathology; Bacteriologist of the Medical Department, University of
Texas, Galveston, Texas.
The room used for all these investigations is a small one, con-
taining 850 feet of air space, situated on the second floor of the
Medical College building. It has one window, 3x4 feet, about 8
feet from the floor, and one large door. ' All the noticeable cracks
and crevices were pasted over with strips of paper, other places were
stuffed with old rags; the cracks about the door made as air tight
as possible with cotton. Even after stopping the room in thi£
manner, it presented the following difficulties in securing the best
results. 1. It is small, and so a small charge of alcohol is to be
used. It requires the same amount of alcohol to heat the asbestos
disc, when a small amount of alcohol is used as when a large
quantity is taken, so, in a small room, a comparatively greater
amount of the alcohol is used up before the generation of formal-
dehyde begins. 2. A number of large water and steam pipes pass
through the floor and ceiling of the room and it is difficult to pack
the openings about the pipes airtight. 3. No way of withdraw-
ing the infected material was had save by opening the door, and
as this had to be done seven times in the twenty-four hours, it will
be seen that a great diminution of the gas must have occurred.
That the room leaked, was evidenced by the strong odor of formal-
dehyde which prevailed in the corridors and rooms during the
experiments.
Four pathogenic organisms were used, the streptococcus pyo-
genes, bacillus typhosis, bacterium anthracis, and mycobacterium
diphtheriae. These organisms represent a fair range in their
resistance to destructive agents, the anthrax being most resistive.
In each series, three conditions of exposure were prepared. The
first was made~by dipping bits of sterile glass in a bouillon culture
of the organism, placing it on a bit of sterile cotton and covering
it with a very loose bit of cotton. This gave a practically free
exposure, with great danger of contamination subsequent to expo-
sure to the gas. In the second, we dipped bits of sterile filter paper
in the bouillon cultures and placed them between two layers of a
felt (billiard cloth), weighing 367 grams per square meter. This
felt is quite compact and is more difficult of penetration than the
ordinary blankets. The third condition was like the second, save
the paper was protected by two thicknesses of felt.
Also in each series (with the exceptions shown in the tables),
inoculations were made at stated intervals into sterile peptone and
bouillon tubes. These intervals are, at the close of the generation
of the gas (usually one hour after starting), 2, 3, 6, 12, 18 and 24
hours. These inoculated tubes were incubated at 37 degrees C.
(body temperature), and records were made at the end of 24, 48
and 72 hours. The fluid in the generator was methyl alcohol, to
which 1 per cent carbolic acid had been added.
Series five was done first, and must be regarded in the light of a
preliminary investigation-to determine whether the disc was packed
properly. The results of this experiment were not satisfactory, so
it was repeated in series one, after having properly adjusted the
packing of asbestos.
In series five, on April 10th, the generator was charged with
1500 cc. of the alcohol mixture, 750 cc. of water, with a tempera-
ture of 50 degrees C., were placed in the water pan and the appa-
ratus started in the usual manner. From the first, it was noticed
that the volume of gas usually obtained was not being given off and
the results bore this out. Twenty-four bouillon cultures were used
to infect the glass and filter paper. The anthrax culture appar-
ently contained no spores, as least none were demonstrated by
staining. Inoculations were made at the intervals given above,
and the tubes were grown three days. The results are shown in
tables A and B. Here it witl be seen that the streptococcus was
killed from the first; the bacillus typhosus was not destroyed
entirely until after six hours; clean tubes not being obtained until
twelve hours from the start. The anthrax was readily killed, the
tube containing the bit of glass exposed twelve hours being infected,
but by a coccus which must have gotten in subsequent to disin-
fection. At eighteen hours, the tube containing the filter paper,
covered by two thicknesses of felt, was infected, but by a rod-shaped
organism, too small and thin for the anthrax. All the tubes sup-
posed to contain the mycob. diphtheriae were contaminated, even
after twenty-four hours exposure to the gas. This, however, was
not the diphtheria germ, but has been identified by us as one form
of the B. subtilis. (For further discussion, see on series four.)
In series one, the disc had been properly packed and the amount
of gas given off was satisfactory. Fifteen hundred cc. of alcohol
were again used, 750 cc. of water, at 50 degrees C., placed in the
evaporating pan. That the air was pretty thoroughly saturated
was evidenced by the deposition of moisture on the window pane.
Exposures were made as before, the material being taken from
24-hour bouillon cultures. By staining, no spores were demon-
strated in the anthrax. By reference to tables C and D, it will be
seen that complete destruction of the streptococcus pyogenes was
effected at the end of two hours; with the B. typhosus this was
done in three hours; with the Baet. anthracis, at the end of three
hours; the tube containing the bit of glass was contaminated- at the
end of forty-eight hours. It is to be noted, however, that all the
2-hour tubes were clean. The Mycob. diphtheriae was destroyed
all the way through after two hours.
In series two, 1000 cc. of fluid, were used, and 750 cc. of water
at 50 degrees C. At the end of twenty-four hours, 250 cc. of this
water was still unevaporated. Spores were present in the culture
of anthrax; 48-hour cultures being used. Inoculations, as before,
were incubated. Tables E and F will give the results. All the
streptococci tubes were clean at three hours; one of the 3-hour
typhoid tubes was infected in seventy-two hours, but by a non-
motile, rod-shaped organism too thick to be typhoid, all the typhoid
bacilli being killed in three hours. The anthrax was killed in six
hours this time, while the diphtheria was killed all the way through.
In series three, exposures were made under similar conditions,
save here 750 cc. of alcohol and 750 cc. of water, at 50 degrees C.,
were used. Of this water, there remained at the end of twenty-
four hours 400 cc. Again, inoculations were made at the stated
intervals. Here (tables G and H), two hours were sufficient to
destroy all the streptococci. The tube containing the bit of glass
exposed for six hours is excluded, since it contained no cocci, but a
rod-shaped organism, undoubtedly due to external contamination.
For the B. typhosus, to be sure of its destruction, an exposure longer
than three hours is necessary for this amount of fluid, since one
tube was infected at the end of three hours. The same time is
necessary for the Bact. anthracis, its lethal time for this amount
of alcohol probably being somewhere between three and six hours.
All the tubes of the Mvcob. diphtheriae were clean thus substan-
tiating the statement of the authorities as to the easy destructibility
of this germ.
In series four the generator was charged with 1500 cc. of fluid
and 750 cc. of water at 50 degrees C., and operated as in series one,
except this time preparations, were made of a thick, rod-shaped
organism (identified as the bacillus subtilis, the same that con-
taminated the Mycob. diphtheriae in series five), and also bits of
filter paper were dipped into cultures of the four organisms used
before, these then sealed in envelopes and distributed, as follows:
-One placed in the inside pocket of a woolen coat, hanging six feet
from the floor, another in the pocket of a pair of woolen trousers,
-also hanging up; a third was laid, unprotected, on top of the book
case (four and one-half feet high); the fourth pinned to the wall,
-eight feet high and just under the only window. These envelopes
remained in the room the full time of the experiment (twenty-four
hours), and the bits of paper were then inoculated irn.o bouillon
and incubated for seventy-two hours. At the end of this time,
only one tube was infected, that containing the anthrax culture,
exposed by placing in a sealed envelope in the inside coat pocket.
These results may be seen in table L The tubes inoculated with
the B. subtilis were all contaminated at the end of seventy-two
hours incubation, even those exposed to the gas for twenty-four
hours, thus indicating a high resistance. Spores in the original
culture were demonstrated by staining.
In this experiment, estimations of the percentage of gas were
made by aspirating the air of the room through a standard ammo-
nium hydrate solution, then titrating this with a standard hydro-
chloric acid solution to estimate the loss of the ammonia combining
with the formaldehyde. At the end of one hour, from the time
the generator was started, there was 1.6 per cent of gas present
• (this by volume) ; at the end of three hours, 95 per cent; at the
end of four hours, .75 per cent. This marked diminution in the
percentage is probably due to the leakage, the escape of the gas in
opening the door to remove the infected material, and by going in
solution in the moisture present. It is to be recalled that the
results obtained* by this method have been shown by observers to
be only approximate.
CONCLUSIONS.
From these trials it would seem that 1000 co. of alcohol are suffi-
cient to effectually destroy the organisms employed in three hours
time in a room containing 850 eubic feet of air space, or when gen-
erated in the proportion of 1.6 per cent by volume or above. It
would probably be best if the charge be so arranged as to give
approximately 2 per cent by volume in rooms having more aper-
tures than the one used, and it may be surmised that in rooms
larger than this, where a greater proportion of the alcohol is avail-
able for gas generation, the charge would be approximately 1000
cc. for each cubit foot of air space.
Excluding the instances in the report and appended tables, where
evident contamination has occurred after disinfection, it may
safely be said that, at least with the same conditions used in these
experiments, the gas generator is efficient in the destruction of the
streptococcus pyogenes, the B. typhosus, the Bact. anthracis, the
Mycob. diphtheriae, in from three to six hours of exposure direct
or no more direct than described above. Its high rate of efficiency
is evidenced by its distinct destruction of the sporulating anthrax
under the conditions named. That, however, there are exceptional
organisms capable of resisting as full exposure to formaldehyde as
herein named, is proved by the type of B. subtilis-contaminating
the diphtheria in series five, and-later, after identification, the sub-
ject of special experiment in series four (table I). This surpris-
ing result is not without precedent, as special forms of this organ-
ism have been found to resist streaming steam for sixteen hours
(Bosenau, in “Disinfection and Disinfectants/’ p. —).
Allen J. Smith,
Professor of Pathology.
James J. Terrell,
Demonstrator of Pathology.
SERIES FIVE—TABLE A.
1500 C. C. of Alcohol Used.
Bac- Time of	Condition 24 hrs. 48 hrs. 72 hrs. Ramarks
terla. Exposure. of Exposure. 37® C. 37® O. 37® C. w-emarKs.
Glass bits in very ....................
loose cotton.____________________________________________c
Filter paper covered .................. "
generation	t>y one layer of felt._________________________________
Filter paper covered ..................
_________________by two layers of felt._________________________________
Glass bits...;...	. ...... .........................
g 2 hours	One layer....... ..... ...... Z=ZZZZZZZZ
g ______________ Two layers..........	. ...... — .______________________
g	Glass bits................... .........................
3 hours	One layer.......	. ...... ......
m ______________ Two layers..........	. ...... .........................
g	Glass bits...... ..... ......	......................
g 6 hours	One layer....... ..... :..... .........................
§____________________Two layers......	. ...... .........................
a	Glass bits...... ..... ...... .........................
£	12 hours	One layer....... ..... ...... .........................
®____________________Two layers......	. ...... .........................
Glass bits...... ............ .........................
18 hours	One layer............. .	...
____________ Two layers..........	. ...... .........................
Glass bits...... ....;.	. .........................
24 hours	One layer............. ...... ......
_________________Two layers..,...	. ...... .........................
~	Glass bits...... ..... ...... .........................
Aseneration One layer............ ..... -I-	-|- Motile bacillus.
_________________Two layers...... ■ -|-______-|-__>|- Motile bacillus.
Glass bits...... ............ .........................
2	hours	One layer....... -|-	-|-	-|- Motile bacillus.
____________  Two layers.........	-|-____-|-	-|- Motile bacillus.
Glass bits...... ..... ....... ........................
3	hours	One layer.................... .........................
g ___________________ Two	layers........ -|-	-I-	Motile	bacillus.
g	Glass bits...... ..... ...... _________________________
6 hours	One	layer.... ..... ....	-|-	Motile	bacillus.
►> __________________ Two	layers...	. -[-________-|-	Motile	bacillus.
Glass bits...........................       ;__________
ctj 12 hours	One layer....... ..... ....... ..... ..................
_________________Two layers......	......	. ...... ............._____
Glass bits...... ..... ...... .........................
18 hours	One layer....... ..... ...... ....................
_________________Two layers......	. ...... .........................
Glass bits...... ..... ...... .........................
24 hours	One layer....... ..... ...... .........................
Two layers............ .............
... Tubes uninfected. -1- Tubes infected.
SERIES FIVE—TABLE B.
1500 C. C. of Alcohol Used.
Bac- Time of	Condition . 24 hrs. 48 hrs. 72 hrs. Ramarks
teria. Exposure. of Exposure. 37® O. 37" 0.	37° C.
Glass bits in very ......................
loose cotton,___________________I_________________________
At close of Filter paper covered ...................
generation, by one layer of felt._____________________________________
Filter paper covered ........... ........
	by two layers of felt.______________
.______________________________________________________Glass bits. .
cn--------------------------------------------------------------------------
®	2 hours	One layer....... .... ....... .........................
g____________________Two layers......	.. ....... .........................
“	Glass bits...... .... ....... .........................
Z 3 hours	One layer....... ..... ...... .........................
.	_________'_______Two layers......	.. ....... .........................
•2	Glass bits...... ..... ...... .........................
g 6 hours	One layer....... ......... .. .........................
5____________________Two layers......	... ...... ......______________-
Glass bits...... -|-	-|-	-|- Coccus.___________
.	12 hours	One layer.......	... ...... .........................
g____________________Two layers......	.. ....... .........................
M	Glass bits.......	... ...... ....................'
18 hours	One layer....... .... ....... .........................
Two layers...................... -|-	Bacillus (rod) not
_____________ the anthrax.
Glass bits.................................................       '
24 hours	One layer .............. ....	......................
_________________________Two layers......	....	.. ......
Glass bits...... -|-	-|-	-|- (See report.)
At close of	One layer....... -|-	-|-	-|- B. Subtilis.
______________Two layers..................-|-	-|-	-|-_______________
Glass bits........................................................... -|-	-|-	-|-_
.	2 hours	One layer....... -|-	-|-____-|-__________.
8____________________Two	layers......	-|-____-|-____-|-__________
®	Glass bits........ -|-	-|-	-|-___________________
5	3 hours	One layer....... -|-______~|~____-|-___________________
a.___________________Two layers......	-|-____-|-____-I-________________  .
C	Glass bits.............   -|-	-|-___________________
8	6 hours	One layer....... -|-	-|-	-|-____________________
5____________________Two layers........	-1-	-|-	-|-___________________
g	Glass bits...... -|-	-|-____-|-___________________
g 12 hours	One layer....... -|-	-|-	-r|-__________________
■g___________________Two layers......	-|-	-|-	-|-___________________
Glass bits...... -|-	-I-____-I-__________
3	18 hours	One layer....... -|-	-I-	-|-___________________
_________________Two layers......	-|-____-|-	-|-______
Glass bits...... -|-	-|-	-|-____________________
24 hours	One layer....... -|~	-|-____-|-___________________
Two layers......	-|-	-|-	-|-
... Tubes uninfected. -|- Tubes infected.
SERIES ONE-TABLE C.
1500 C. C. of Alcohol Used.
✓	_	_	a.
Bac- Time of	Condition 24 hrs. 48 hrs. 72 hrs.
teria. Exposure.	of Exposure. 37® C. 37® 0.	37® 0. ■
Glass bits in very ......	............
loose cotton.______________________'_______
At close of Covered by one layer ................... -|- Streptococcus,
generation, of felt._________________>___________________
Covered by two .................. -|-	-|- Streptococcus.
layers of felt.____________________________
Glass bits........... ... ....... .......
2 hours	One layer................ ...............
® ----------------------------£------------------------------------------------------
c______________________Two layers...........	... ....... .......
be	Glass bits............... ....... .......
O------------------------------------x.— --------------------------------------------
>>	3 hours	One layer............ ... ■ ......	...
___________________Two layers...........	... ....... .......
g	, Glass bits............. ......	... ....................
g 6 hours	One layer............ ... ....... ....
g______________________Two layers...........	... ....... .......
a	Glass bits........... ... ....................................
£	12 hours	One layer............ ... ....... .......
rn	Two layers...........	... ....... ............................
Glass.bits.............	. ....... ............................
18 hours	One layer............ ... ....... .......
Two layers...........	... ....... ............................
Glass bits........... ... ....... ............................
24 hours	Onje layer........... ... ....... ............................
___________________Two layers...........	... ....... | ..........................
Glass bits in very ..........................
loose cotton.	____
At close of Covered by one layer ................... -|- Rod shaped—
generation, of felt. ________________ _______________________motile.__________
Covered by two .................. -I-	-|- Rod shaped—
layers of felt.________	■■______________motile.__________
Glass bits...-.iTO-..■",./&£'	.....	_>_______________
2	hours	One layer............	.....	......	........................
Two layers................... -|-	-|- Rod shaped—
__________________motile._________
Glass bits........... ... ....... ....... ....................
3	hours	One layer............ ... ....... ....... ....................
g______________________. Two layers.........	... ....... ....... ....................
Glass bits.............	. ....... ............................
6 hours	One layer...............   ....;■	........................
.	____________ Two layers.............. ... ....... ............................
®	Glass bits........... ... ....... ............................
12 hours	One layer............ ... ....... ............................
Two layers...........	... ....... ............................
Glass bits........... ... ....... ............................
18 hours	One layer............ ... ....................................
Two layers..........	... ....... ............................
Glass bits........... ... ....... ......______________________
24 hours	One layer............ .;....	.......	_
Two layers...............................
... Tubes uninfected. -|- Tubes Infected.
SERIES ONE—TABLE D.
1500 C. C. of Alcohol Used.
Bac- Time of	Condition 24 hrs. 48 hrs. 72 hrs. Remarks
teria. Exposure. of Exposure. 37° O. 37° O. • 37® C.
Glass bits in very •............. -|- Rod shaped.
\	loose cotton.__________________________
At close of	Covered by one layer  	z	-|- Rod shaped,
generation. Of felt._________________________________
Covered by two ................ -I-	-|- Rod shaped.
a	layers of felt,_______________.	‘,	____________
g	Glass bits........	......... . ........ .
§•	2 hours	One layer....... ........... . ........ ..............
o___________________Two layers......	......... . ........ ..............
Glass bits............   -I-	-|~ Rod shaped.
u5 3 hours	One layer....... ........... . ........ .......
o	Two layers.................... ——
g	Glass bits...... ........... . ........ '	_______
g 6 hours	One layer...,... .... ........ ........
■5	Two layers......	....'.	.. .......... ..............
E	Glass bits...... ........... . ........
5	12 hours	One layer..............................
Pm	---------— -------------------------------- --------—■
<p__________________Two layers......	.....     .........................
§	Glass bits..................................       ~
ffl 18 hours	One layer....... .... ...... ..........
_____________ Two layers...,..... .................... . __
Glass bits........................................... 	     "~ZZ~~ZZZZZ
24 hours	One layer...... ...... ...... ...................
_____________________ Two layers		. . .......................
Glass bits.....................................................................        ______
AtgCenwa°tion.__Onela?er........ .... -----------±- Irregular rods^_
I___________ Two layers.........	... -|-	-l~ Irregular rods.
Glass bits...... .... ...... ..........
2	hours	One layer.......	.... ........ ____________
8	____________ Two layers.........	......... . .......................
®	Glass bits...... ......	..... .......
5	3 hours	One layer.................................         _
a.__________________Two layers......	......... . ........ •_____________
q	Glass bits...... .... ...... ...........................
g 6 hours	One layer...........   ...a.	...... ..............
5	____________ Two layers.........	.. ...... ..................__........
®	Glass bits...... ........... . .......____________
g 12 hours	One layer....... ........... . '......______ _________-
•g__________________Two layers......	... ..... ...........................
£	Glass bits...... ........... . ........___________
g 18 hours	One lay er......	.. ...... ......____________________
________________Two layers		. . .... .................._
Glass bits............................................................ . . ....
24 hours	One layer....... ......	... .................
Two layers.............................
...Tubes uninfected. -|- Tubes infected.
SERIES TWO—TABLE E.
1OOO C. C. of Alcohol Used.
Bac- Time of	Condition 24 hrs. 48 hrs. 72 hrs. Rpmarifu
teria. Exposure.	of Exposure. 37° 0.	37® 0.	37® 0.
Glass hits in very ......................
loose cotton.____________________________
At close of Covered by one layer .....................
generation. of felt,	J_________________________
Covered by two .................... -|-	A coccus.
_________________layers of felt.______________________
Glass bits...... ..... ...... .........................
co 2 hours	One layer...................     -|-	A coccus.______
a____________________Two layers......	... ...... -|- A coccus.
»	Glass bits.............. ..;.........
o	-----------------------------------------------------------
>»	3 hours	One layer....................      ,___________________
w____________________Two layers......	... ...... ...................
§	Glass bits........   I	.. ....... |________________
o 6 hours	One layer...... ......	... .........................
o____________________Two layers........	... ...... ......	..
a	Glass bits...........................
Q	-----------------------------------------________________
5	12 hours	One layer.......................................       ,
00___________________Two layers......	......	... .........................
Glass bits....... .... ...... .........................
18 hours	One layer......................................        .
__________________Two layers......	... ...... ...... ..................
Glass bits............................................................... . . ..
24 hours	One layer...............     <	......................
_________________________Two, layers.....	......	... .........................
Glass bits...... .. ...	... .........................
At close of	One	layer............. -|-	-I-	Motile bacillus.
- n ra OD'	Two layers......	  -|-_____-I-	Motile bacillus.
Glass bits........	... ...... .........................
2	hours	One	layer..........   -|-_____-|-	Motile bacillus.
_________________Two layers......	......	-|-____-I-___________________
Glass bits...... ..... ...... .........................
3	hours	One layer....... ..... ...... | ..... .......
s	Two layers....................   -|-	Non-motile, rod
o	shaped to thick
_____________ ___________________________________________for typhoid.
►>	Glass bits...... ..... ...... ...................
m 6 hours	One layer....... .....	... .........................
5	_____________ Two layers.........	... ...... .........................
12 hours	Ope layer...............   .	...	......................
_________________Two layers.......	.......... ...................
Glass bits........................................................ . . ..
18 hours	One layer....... ..... ...... .........................
__________________Two layers................ .. ...........................
Glass bits................................................................. 	 . ..
24 hours	One layer...... ......	....	......................
Two layers............ ............
...Tubes uninfected. -I- Tubes Infected.
SERIES TWO-TABLE T.
1OOO C. C. of Alcohol Used.
Bac- Time of	Condition 24 hrs. 48 hrs. 72 hrs. Rnmarks
teria. Exposure.	of Exposure. 37° C. 37° C. 37° 0.
Glass bits in very ......................
g	loose cotton.___________________________________
•g At close of Covered by one layer ........ -|-	-|-	Rod shaped^
•g generation. of felt._______________________________________________________
®	Covered by two ............ -|-	-|-	Rod shaped.
>>	___________ layers of felt.____________________________________
o	Glass bits........ .... ...... .................
®	2 hours	One layer.......... ... ...... ......._________
©____________________Two layers........	....____-|-____-|- Rod shaped.
a	Glass bits........ .... ...*»	.............'________
”	3 hours	One lay er........ .... ......	...
o *__________________Two layers-.rr;..?.. .. -|-_ _-I-	Rod shaped.
m	Glass bits........■	... ...... ......___________~
6 hours	One layer......... .... .............. '
”	____________ Two layers..........	... ...... ....... ~
g	Glass bits...i....	... ...... .....................'
5	12 hours	One layer......... .... ...... .......
o____________________Two layers........	... ...... ....... ~
Glass bits....... .... ...... .......
§	18 hours	One layer.......................................       '
g _________________ Two layers.........	... ...... .......
•g	Glass bits........ .... _____	....
5	24 hours	One layer......... .... ...... .......
_________________________Two layers........	... ...... ....... ‘
Glass bits....... .... ...... ................., -______
At close of	One layer................................
■generation. Two layers..........“777	777	777
Glass bits....... .... ......	... ..................
2 hours	One layer......... .... ...... ....... ...........
gj_______________Two layers........................ ..........................
"5	Glass bits............. ............
©-----------------------------------------------------------------------------
■o 3 hours	One layer......... .... ...... ..........................
o<	Two layers........	... *..... .....................
Q	Glass bits........ .... ...... ..........................
8	6 hours	One layer......... .... ...... .....................
•g___________________Two layers........	... ...... ..........................
®	Glass bits........ .... ...... .....................
cs 12 hours	One layer......... .... ............	_________
o____________________Two layers........	... ...... ..........................
t»	Glass bits.........	... ...... ..........................
*	18 hours	One layer....... ......	.. ......____________________
_____________| Two layers.......... .... 777	... .............
Glass bits....... .... ...... ....... __________
24 hours	One layer........ ............	... •_________________
Two layers................	............
...Tubes uninfected. -|- Tubes infected.
SERIES THREE—TABEE G.
750 C. C. of Alcohol Used.
Bac- Time of	Condition 24 hrs. 48 hrs. 72 hrs. Romarke
teria. Exposure. of Exposure. 37° C. 37® O. 37® C.
Glass bits in very ........................
loose cotton.__________________________.
At close of Covered by one layer.........................
generation. of felt. J_________________________________
Covered by two -|-	.-|- Streptococcus
_________________layers of felt._____________________________________
Glass bits............................................................ ......	. .
•	2 hours	One layer.......... -|-	-|-	-[- Streptococcus.
®	Two layers........... -|-	-|-	-|- Streptococcus.
®	Glass bits......... .... ....... .......
°^3 hours	One layer....................................     .
Two layers........	... ......	................
a	Glass bits.................. -|-	-I- Rod shaped. Not
g___________________________________, .____________________________streptococcus.
o 6 hours	One layer....................... .......
o	Two layers.........	... ......	....
~ Glass bits.......... ......	..... .....
£	12 hours One layer................ ........ ...	.... ZZ2ZZZIZZZZI
02	Two layers.........	... ....... .......
Glass bits............... ..... ....... •	_______
18 hours	One layer.......... ■■■■	..... ....... ZZZ
Two layers.......	..... ....... .......
Glass bits........ .... ....... .........
24 hours	One layer.......... .... ....... ....... ~_________________
Two layers........	... -|-________-|- Did not stain._____
Glass bits........,	.......	... ..........................
At close of	One layer................... -|-	-J- Bacillus.
generation. Two layers............	^77	-|-	-|- Bacillus.__________
Glass bits.......	....... ; ... ,	... ._________________
2 hours	One layer..........	... -|-	Bacillus.
Two layers........	-|-	-|-___-|- Bacillus.__________
Glass bits...............      ......
g 3 hours	One layer.......... .... ....... ....... ~
S	Two layers.......................   -|-	Bacillus. _______
x:	Glass bits......... .... ...............
Q,	--------------- —---------------------------------------------
>>	6 hours	One layer..............   ......	.........-__________.
Two layers............. .......	....
s	Glass bits..............   .....	....
=	12 hours One layer................ZZZZ —•••	....................
_	Two layers.........	......	__2i_
’	Glass_bits......... .... ....... ..........................
18 hours , One layer..................   ......	.......................
Two layers..........	.. ....... .......
Glass bits........ ......	.... ...........................
24 hours	One layer.......... ......	..... .......
Two layers.................
... Tubes uninfected. -|- Tubes infected.
SERIES THREE—TABEE H.
750 C. C. of Alcohol Used.
Bac- Time of	Condition 24 hrs. 4* hrs. 72 hrs. Ramirki
teria. Exposure. of Exposure. 37° V. 37® 0.	37® C.
Glass bits in very ........................
loose cotton.______________________________________
At close of Covered by one layer ........... -|-	-I- Bod shaped, thick
generation. offeit,_________________________'________■
Covered by two -|-	-|-	-|- Rod shaped, thick
__________________layers of felt.___________________________________________
Glass bits...............    ,	......•______________
2	hours	One layer......... -I-________-|-____-|- Rod shaped, thick
■2	Two layers........	-|-____-|-____-|- Rod shaped,thick
g	Glass bits........ ...................
3	hours ‘ One layer..................... ?......  .	______
a_____________________Two layers........	... ...... -|- Rod shaped, thick
/	. Glass bits............. .. ...... ...........................
3	6 hours	One layer......... ........... ___________________________
g _______________ Two layers............	............	.
g	Glass bits........ .... ...... ...........................
5	12 hours _____One layer.......	..... .............. '_____________
_____________ Two layers............	... ...... ...................
Glass bits...........   ......	.....................
18 hours	One layer......... .v...	......	.....................
__________________Two layers........	... ...... ...........................
Glass bits........ .... ...... ...........................
24 hours	One layer......... ...._	.. ...................’__
__________________Two layers........	... ...... ...........................
Glass bits........ ... .	. ..........................
At close of	One layer....................
generation.----—------------------------------------------=--------------
______________Two layers...........	. ...... ...........................
Glass bits...................      ....___________________
2 hours	One layer................    '	.....................
•___________________Two layers........	... ...... .......
■ -	Glass bits.......	..... ...... ...........................
®	3 hours	One layer......... .... ...... .......
•g____________________Two layers........	... ....... ...... I__________________
£■	Glass bits........ .... ...... ...........................
Q 6 hours	One layer......... .... ...... ......................
3	_____________ Two layers. —............	. ..........................
•g	Glass bits........	.... ...... ■ ......	_________________
S 12 hours	One layer...........  ;	.<,	...J.
2_____________________Two layers........ .... ......	.....................
o	Glass bits........ .... ...... .......
>.	18 hours	One layer......... ......	. ..........................
s_____________________Two layers............. ...... ...................
Glass bits........ ...................
One layer.........:...	......	?	-I- Bod shaped. Not
24 hours	the diphtheria.
Two layers.............'................
...... Tubes uninfected.	-|- Tubes infected.
SERIES FOUR—TABLE I.
1500 C. C. of Alcohol Used.
Bac- Time of	Condition 24 hrs. 48 hrs. 72 hrs. _	,
teria. Exposure. of Exposure. 37° 0.	37® 0.	37® 0. Remarks.
Glass bits in very -|-	-|-	-|-
...	. loose cotton.
At ClOSe Of ------------5-r-----=----------:------;------;-----------------------
generation. Covered by one layer -|-	-|-	-|-
1 a hours	°f felt.__________________________________._______________________
Covered by two -|-	-|	-|-
___________________layers of felt.________________
Glass bits.........	-|-____-|-	-|-
2^ hours	One layer...........	-|-___-|-	' -|-
____________________Two layers......................-|-.-|-	-|-
Glass bits................................................ 	 -1-	-|-
4	hours	One layer.......... -|-	-|-	-|-
___________________Two layers.........	-|-	-|-_____-|-_
Glass bits......... ..... -|-	-|-_____________________
18 hours	One layer.......... -|-_______-|-	-|-_____________________
___________________Two layers..................-I-......-I-_-I-.............
Glass bits.................................................................. 	 -|-	-|-_
24 hours	One layer.......... ..... -|-	-|-_____________________
Two layers.............. -|-	-|-
....Tubes uninfected. -|- Tubes infected.
EXPOSED 24 HOURS IN SEALED ENVELOPES.
Bacteria. Condition of Exposure,	370^0^' ~37«'cS'
In inside pocket of coat.. ..... ....... ........ ....................
x	In pants pocket.......................................................
rep ococcus Lying on top of book- ...................................................
Pyogenes. case.______________________________________________________________________
Tacked on wall 8 feet ..................... ..........................
____________________high._____________________________________.......
In inside coat pocket................................................	. . . ........ .
...	In pants pocket........................ ..............................
Lying on top of book- ................................................
Typhosus. case.______________________________________________________________________
Tacked to wall 8 feet .....	..........................................
'_________________ high._________________________________________________________________
In Inside coat pocket......	...	. -|- Anthrax._________________
_	.	.	In pants pocket...............	~	. ..................................
Paying on top of book- .............................................
Anthraci s.	case._________________________________________________________________
Tacked to wall 8 feet ................................................
____________________high.......................
In Inside coat pocket..........................	... ....... ........ ____________________
,	.	. In pants pocket....................	...............................
Mycobacterium —f--------------------—-—;--------------------------------------------------
Lying on top of book- ............'	....................................
Diphtherias. case.____________________________________________________________________
Tacked to wall 8 feet ................................................
above floor.
......Tubes uninfected.	-|- Tubes infected.
				

