# A Provoking Error

**Published:** 1903-09

**Authors:** 


A PROVOKING ERROR occurred in Dr. Allen J. Smith's article in the August number, "Experiments to Determine the Disinfecting Value of the Germicide Gas Generator." On page 45, tenth line, for "each cubic foot of air space" read "for each 1000 cubic feet of air space."

